# MRi of the knee compared to specialized radiography for measurements of articular cartilage height in knees with osteoarthritis

**DOI:** 10.1016/j.jor.2021.05.014

**Published:** 2021-05-12

**Authors:** Jacob Fyhring Mortensen, Kristian Breds Geoffroy Mongelard, Dimitar Ivanov Radev, Andreas Kappel, Lasse Enkebølle Rasmussen, Svend Erik Østgaard, Anders Odgaard

**Affiliations:** a Department of Orthopaedic Surgery, Copenhagen University Hospital Herlev-Gentofte, Kildegårdsvej 28, DK, 2900, Hellerup, Denmark; b Department of Radiology, Copenhagen University Hospital Herlev-Gentofte, Kildegårdsvej 28, DK, 2900, Hellerup, Denmark; c Orthopaedic Research Unit, Aalborg University Hospital, Hobrovej 18-22, DK, 9000, Aalborg, Denmark; d Department of Orthopaedic Surgery, Vejle Hospital, Kabbeltoft 25, DK, 7100, Vejle, Denmark; e Department of Orthopaedic Surgery, Aalborg Universitetshospital, Hobrovej 18-22, DK, 9100, Aalborg, Denmark; f Department of Orthopaedic Surgery, Rigshospitalet Copenhagen University Hospital, 2100, Copenhagen Ø, Denmark

**Keywords:** Knee, Osteoarthritis, Skyline, Rosenberg, Stress radiography, Varus, Valgus, Stress, Joint space width, Reliability, Agreement, Radiography, Magnetic resonance imaging, JSW, Joint space width, mJSW, minimal Joint space width, MRi, magnetic resonance imaging, mUKR, Medial Unicompartmental Knee Replacement, OA, Osteoarthritis, TKR, Total Knee Replacement

## Abstract

This study aims to evaluate and compare extremity-MRi with specialized radiography by measuring articular cartilage height in patients with knee osteoarthritis.

A prospective study, including sixty patients. Measurements on MRi images, Rosenberg view, and coronal stress radiographs were performed. MRI was compared to specialized radiography.

Measurements in the medial compartment showed negligible/weak correlation between MRi and Rosenber/varus stress. In the lateral compartment, MRi and the Rosenberg/valgus stress view were strongly correlated.

We conclude that MRi cannot replace radiographs for the measurement of articular cartilage thickness. MRi should, however, be reserved for more unusual cases of atypical clinical findings.

## 1 Introduction

To ensure the correct knee implant for the right patient, it is essential to know how different imaging types discriminate between the levels of degenerative disease in the different compartments. To support the correct choice of imaging technique, it is essential to know whether levels of degenerative disease are better visualized using magnetic resonance imaging (MRi) compared to specialized radiography, such as the Rosenberg view and coronal stress radiography.

MRi has long been considered the gold standard for evaluating soft tissue, articular cartilage, and early osteoarthritic (OA) changes. Still, the usefulness in detecting severe OA is less clear.^1^ In clinical practice, MRi is generally not included in the decision-making process when considering knee arthroplasty surgery. It has been criticized in the work-up for knee replacements due to the over-estimation of knee pathology, pricing, and time consumption in healthcare systems where cost efficiency is essential. Although some areas of the world see an increasing use of MRi for endstage knee OA, this possible rise in costs could be minimized when using an extremity-MRi scanner, which is cheaper. Though, this often is accompanied by imaging with lower field strength and resolution on the acquired images.

Conventional radiography of the knee is considered sufficient when offering a total knee replacement (TKR), but complementary specialized radiographs may be necessary when considering a medial unicompartmental knee replacement (mUKR).^2^, ^3^, ^4^ Specialized radiography visualizing the tibiofemoral compartment includes the Rosenberg view and coronal stress radiography, recommended in various radiographic algorithms.^5^^,^^6^ This study set out to compare some of these specialized radiographic techniques with proton density fast spin-echo (PD-FSE) MRi in patients undergoing either a mUKR or TKR.

Only limited information is available regarding articular cartilage height measurements using MRi, and studies comparing MRi with specialized radiography are scarce. This type of study can provide the essential information needed in choosing the optimal diagnostic tool to diagnose the specific type of knee osteoarthritis present, potentially avoiding excess radiation and extra costs in the diagnostic process.

This study aimed to assess the interrater agreement of MRi in a cohort of patients with knee osteoarthritis and compare if MRi and specialized radiography can determine the tibiofemoral joint space width similarly.

## 2 Patients and methods

Guidelines for reporting reliability and agreement studies (GRRAS) were followed^7^ in this prospective diagnostic study. The “STROBE” statement and guidelines were followed.^8^

### 2.1 Patients and population

**Fig. 1:**
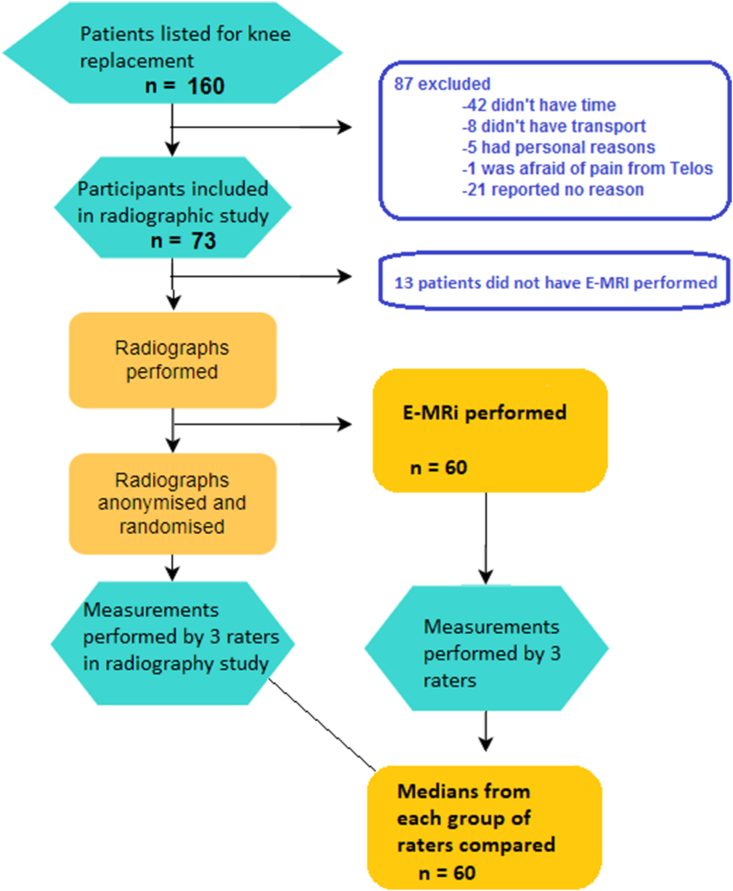
Flowchart.

One hundred and sixty patients were asked to participate in this study at a high-volume knee arthroplasty centre. Sixty patients participated in this substudy between October 2018 and June 2019. Participants had both an MRi scan and specialized radiography of the knee. Eighty-seven patients declined participation, and thirteen only had radiographs performed due to delay of technical setup of the MRi. All sixty patients participated in a separate radiographic study investigating the reliability and agreement of articular cartilage height measurements with the 45° Rosenberg view and 20° coronal stress radiographs. Thirty-three were planned for TKR, and twenty-seven were planned for mUKR (twenty-nine females and thirty-one males). A patient flowchart can be seen in Fig. 1.

The only inclusion criterion was being planned for either a TKR or a mUKR at the enrollment time. The patients selected for inclusion were random and unselected, forming a convenience series depending on the radiographic ward's capacity. Exclusion criteria were pregnancy, severe systemic disease, employment at the department, a lack of ability to comply with simple instructions, and common contraindications for performing MRi, such as having a pacemaker or other metallic implants.

### 2.2 Methods of testing

#### 2.2.1 Radiographs

One experienced radiographer performed all radiographic examinations, using a Siemens Axiom Luminos dRF with fluoroscopy (Siemens Healthcare GmbH, Erlangen, Germany). The focus was set to Fine, with an opening of 0.6 mm. A sequence of radiographs was performed for each patient, consisting of the Rosenberg view, followed by coronal stress in varus and valgus (performed in a twin study, pending publication).

#### 2.2.2 MRi

An Optima MR 430s 1,5 T extremity MRi scanner was used for this study. The patients were positioned in a chair in a reclined position outside of the scanner. Only the patients’ knee was placed inside the scanner. The knee was positioned in the coil with 0–5° flexion, without weight-bearing, as this has proven to show similar cartilage thickness as weight-bearing MRi.^9^ The knee protocol includes proton density (PD) weighted sequence with and without fat saturation in coronal and axial plains. Technical information for Coronal/Axial planes: Slice thickness 3.5/4.5 mm; Gap - 1/1.5 mm; Matrix - 512 × 512 for both planes; FOV - diameter 160mm/150 mm; TR - 1652/1682 ms; TE - 21.8/22.2 ms; NEX - 2 for both planes.

### 2.3 Methods of assesment

#### 2.3.1 Radiographic measurements

**Fig. 2:**
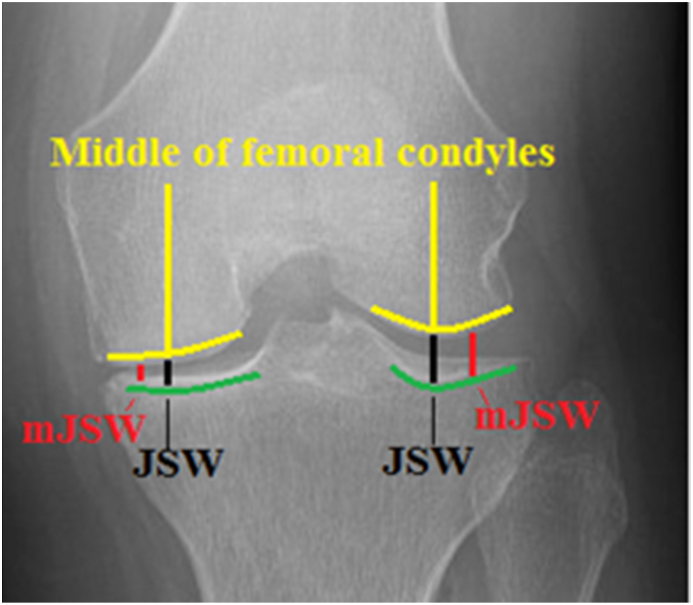
Radiographic measurements of JSW and mJSW in specialized radiographs.

Magnification calibrated, anonymized, and randomly ordered measurements of the radiographs were used from three individual raters, consisting of consultant orthopedic surgeons. The median of the three raters’ measurements was calculated for each parameter, using the first of three rounds of measurements. Parameters consisted of central joint space width (JSW) and minimal joint space width (mJSW), measured in millimeters with one decimal in each tibiofemoral compartment for each type of specialized radiograph (see Fig. 2) (performed in a twin study, pending publication).

#### 2.3.2 MRi measurements

Four sets of images on PD-FSE images in coronal and axial planes, with and without fat saturation, per scanned knee, were obtained and reviewed on an IMPAX Site working station 6.6.1.8006 (Agfa-Gevaert, Mortsel, Belgium). Orthopedic resident, a Radiology resident, and a certified radiologist with seven years' experience as a musculoskeletal imaging consultant. The medians of the three raters’ measurements for each parameter were used. Measurements were primarily conducted on the PD-FSE image without fat-saturation, and images with fat-saturation were used to help confirm difficult measurements.

**Fig. 3:**
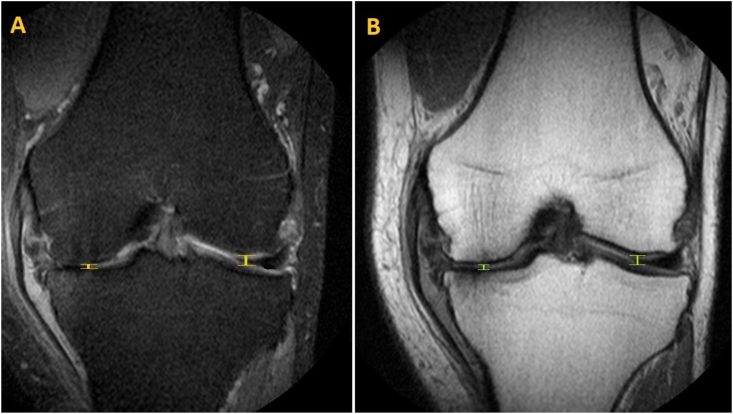
MRi scanning using PD-FSE imaging with fat saturation (A) and without fat saturation (B), showing the standardized central measurement of the articular cartilage height in the tibiofemoral joint medially and laterally, measured as JSW.

Articular cartilage height was measured in both the medial and lateral tibiofemoral compartment. Each knee compartment was assessed by measuring the femoral and tibial bone-cortex distance in the compartment, which was considered the JSW. Measurements were also performed at the smallest distance perceived (mJSW) between the femoral and tibial bone cortex at a weight-bearing location, seen in Fig. 3A+B. No attempt was made at measuring the individual cartilage heights of femoral or tibial surfaces, as this was not accurately feasible due to the low pixilation and slice width.

### 2.4 Statistics

Data presented itself as right-skewed and clustering towards zero. Therefore data used for reliability analysis was rounded to the nearest integer, and non-parametric weighted Cohen's Kappa was used to determine agreement and reliability. SPSS statistical package version 22 (IBM SPSS Inc, Chicago, IL) and Microsoft Excel (Microsoft Corporation, Washington, USA) with Realstatistics-data-analysis-tool add-in were used to calculate linear weighted kappa with 95% confidence interval. Strength of agreement and reliability were categorized as follows; Poor (<0.00); Slight (0.00–0.20); Fair (0.21–0.40); Moderate (0.41–0.60); Substantial (0.61–0.80); Almost perfect (0.81–1.00).^10^ Data used to compare radiographic techniques to MRi measurements consisted of the medians of the three raters' measurements for each measurement type (see flowchart in Fig. 1). Spearman's rank correlation coefficient was used to summarise the strength of the relationship between the radiographic techniques and the MRi measurements and was interpreted as follows; Negligible (0–0.1); Weak (0.1–0.39); Moderate (0.4–0.69); Strong (0.7–0.89); Very strong (0.9–1).^11^ Mean difference and limits of agreement were analyzed using Bland-Altman plots, along with scatterplots to show the strength of agreement between variables.^12^ Comparison of demographic data was made using the Student's T-test. A comparison of means between techniques was made using a paired T-test.

## 3 Results

Demographics of sixty included patients, showed a mean age of patients 71 ± 8 years with no difference between sexes (p = 0.9) and implant types (p = 0.4). The mean BMI was 28 ± 4 kg/m^2^, with no difference between sexes (p = 0.4) and implant types (p = 0.5).

### 3.1 Specialized radiography agreement and reliability

All raters and participants completed the protocol.^13^ In this study (n = 60), mean measurements were calculated for each technique in each compartment. For the Rosenberg view, mean measurements of JSW(SD)/mJSW(SD) were 1.6(1.6)/1.1(1.4)mm in the medial compartment and 6.4(1.9)/5.5(2.0)mm in the lateral compartment. For coronal stress radiography, mean measurements were 1.6(1.7)/1.0(1.3)mm in the medial compartment in varus stress and 5.9(1.6)/5.1(1.7)mm in the lateral compartment in valgus stress.^13^

### 3.2 MRi agreement

Mean measurements of JSW(SD)/mJSW(SD) were 1.9(1.0)/0.9(0.6)mm in the medial compartment, and 4.3(1.6)/3.7(1.5)mm in the lateral compartment. Interrater analysis for MRi showed primarily fair to substantial agreement medially (JSW/mJSW; 0.46–0.64/0.38–0.62) and moderate to substantial agreement laterally (JSW/mJSW; 0.44–0.53/0.46–0.61), which can be seen in Table 1.

**Table 1:**
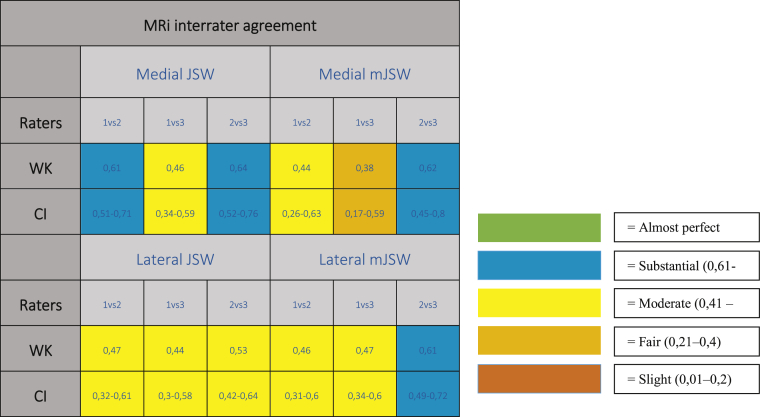
Weighted Kappa, with 95% confidence interval, of MRi interrater agreement in the medial JSW/mJSW and lateral JSW/mJSW.

### 3.3 MRi vs. specialized radiography

#### 3.3.1 Medial compartment

**Fig. 4:**
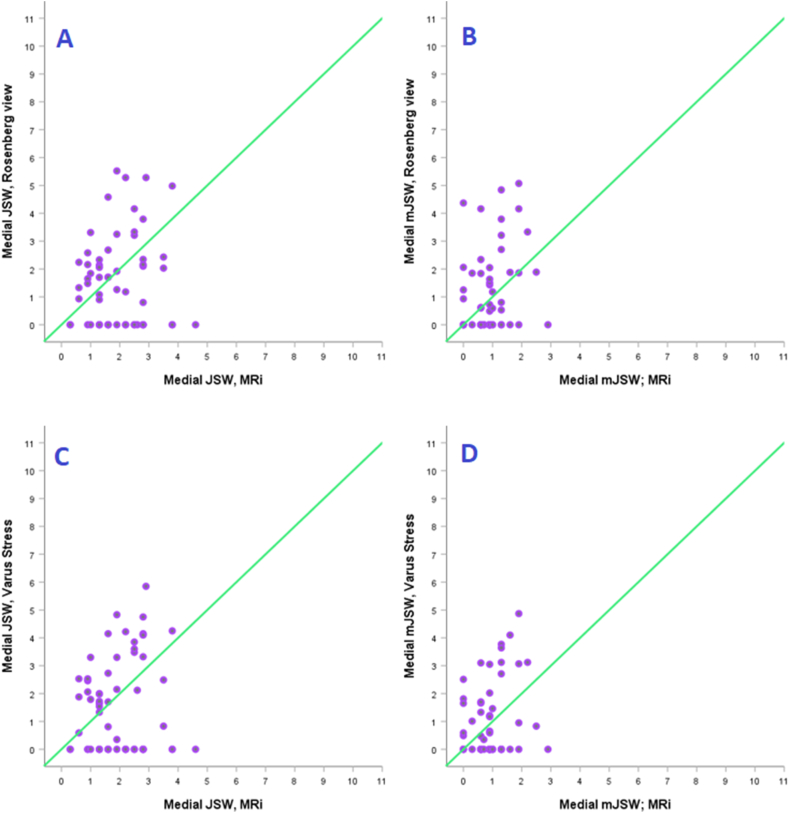
Medial knee compartment - Scatterplots of MRi vs. specialized radiography in measuring JSW/mJSW.

Comparing MRi measurements with the Rosenberg view showed negligible to weak correlation medially (JSW/mJSW; r = 0.07/0.22; CI = −0.17–0.33/-0.06–0.48; p = 0.3/0.042) which was non-significant. Comparing MRi measurements with the varus stress radiography showed weak correlation medially (JSW/mJSW; r = 0.11/0.15; CI = −0.18–0.36/-0.23–0.41; p = 0.19/0.31) which was also non-significant. Scatterplots can be seen in Fig. 4.

#### 3.3.2 Lateral compartment

**Fig. 5:**
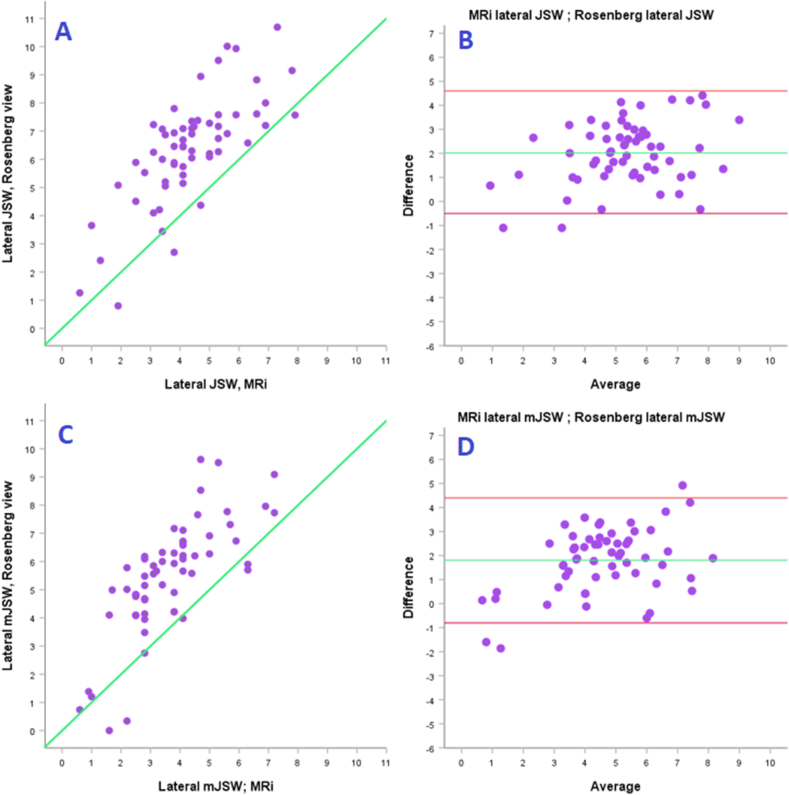
Lateral Knee compartment - Scatterplots (A + C) and Bland Altmann plots (B + D) comparing the Rosenberg view to MRi in measuring JSW/mJSW.

**Fig. 6:**
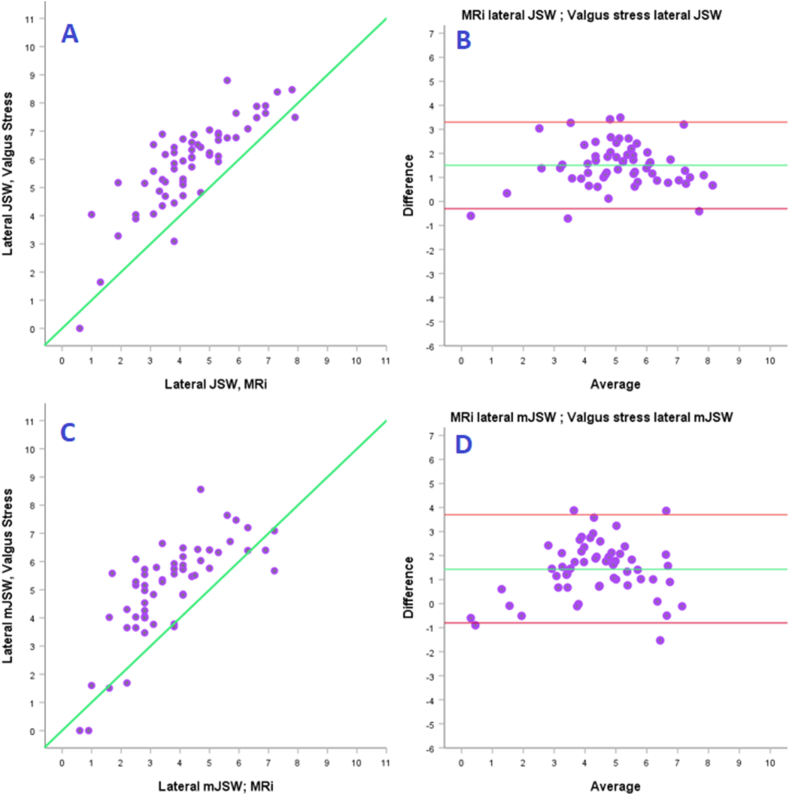
Lateral Knee compartment - Scatterplots (A + C) and Bland Altmann plots (B + D) comparing valgus stress to MRi in measuring JSW/mJSW.

Comparing MRi mJSW measurements with the Rosenberg view showed strong correlation laterally (JSW/mJSW; r = 0.74/0.79; CI = 0.58–0.85/0.67–0.87; p = 0.001) which were both highly significant. Comparing MRi mJSW measurements with the valgus stress radiography showed strong to very strong correlation laterally (JSW/mJSW; r = 0.82/0.77; CI = 0.68–0.90/0.62–0.87; p = 0.001) which were both highly significant. Scatterplots and Bland Altmann plots show systematically and consistently lower measurements with MRi, with a mean difference ranging from 1,5 to 2 mm, and limits of agreement ranging relatively widely (see Fig. 5, Fig. 6).

## 4 Discussion

### 4.1 MRi vs. specialized radiography

#### 4.1.1 Medial tibiofemoral compartment

No significant correlation was found between MRi and specialized radiography when measuring JSW/mJSW in the medial knee compartment. Scatterplots showed that many patients presented with bone-on-bone in the medial compartment with the Rosenberg view and varus stress JSW/mJSW but were measured with several millimeters of cartilage on MRi (see Fig. 4). These larger measurements on MRi were seen more so when using JSW than mJSW. These results could be explained by the knee's position in 0–5° flexion compared to 20–45° flexion using specialized radiographs. Also, the lack of weight-bearing could result in measurements of more than just articular cartilage, but earlier studies of weight-bearing vs. non-weight-bearing MRi have shown this to be of minimal difference.^9^ This is the clinical reality when using MRi. It is typically impossible to have MRi performed with weight-bearing or a higher flexion of the knee due to the coil's placement around the lower extremity. These findings represent a significant concern of MRi's ability to assess cartilage height in late-stage OA knee compartments since half of the study population presented with isolated medial OA. This is further supported by the lack of correlation to the specialized radiographs in the medial compartment.

Altogether, this study's findings show that MRi is not suited for diagnosing bone-on-bone and endstage OA in the medial knee compartment. This can be regarded as a positive finding since it should further discourage using an expensive imaging technique such as MRi for solely to confirm medial bone-on-bone OA. Furthermore, it should be discouraged when bone-on-bone can be assessed sufficiently with a cheap and straightforward radiographic image such as the Rosenberg view or varus stress radiography.^13^

#### 4.1.2 Lateral tibiofemoral compartment

A strong to very strong correlation was found between MRi and specialized radiography in the lateral compartment. Valgus stress measurements compared to MRi (JSW) showed the best correlation (r = 0.82), albeit valgus stress mJSW and the Rosenberg view also with high correlation (r = 0.74–0.79).

When analyzing the scatterplots and Bland Altman plots, we saw that MRi consistently measured 1.5–2 mm smaller JSW/mJSW than both special radiographic techniques. When considering offering a mUKR, full-thickness cartilage should be present.^5^^,^ ^14^ Considering this correlation between techniques and the small difference in measurements between specialized radiographs and MRi, we can conclude that both types of specialized radiography and MRi can be used to confirm full-thickness cartilage in the lateral compartment. This should further discourage the use of MRi for general screening of patient suitability for mUKR in regards to cartilage height assessment.

### 4.2 MRi

We found moderate to substantial agreement between all raters on MRi measurements, except for one category between rater 1 and 3 (Medial mJSW), showing that overall agreement was acceptable. Previous studies have generally proven good inter- and intrarater agreement when assessing the knee's cartilage status.^15^ However, studies are scarce in comparing raters in the measurements of tibiofemoral JSW and mJSW on extremity MRi. Performing test-retest measurements were considered redundant with MRi, as previous studies have shown high reliability, even when comparing with different field strengths.^16^ When reviewing the current literature, no other studies have compared the Rosenberg view and coronal stress radiography with MRi, in a larger population of patients with endstage OA, listed for either a mUKR or TKR. This study has shown that the low tesla MRi is a good tool for visualizing the height of intact cartilage laterally, but that this is similarly done with the Rosenberg view or valgus stress radiography. It also shows that MRi is quite unreliable when assessing cartilage height in significantly deteriorated knee compartments. This should be considered when using MRi for general screening of patients for mUKR, as both of these assessments can be done on specialized radiographs similarly.

### 4.3 Limitations

MRi scanning of the knee joint was performed in the axial and coronal plane due to the study's time and practical limits, but imaging in two planes was considered sufficient for the study. The use of a 1.5 T extremity scanner may present a limitation on the quality upon which the imaging is measured due to larger pixel size and lower definition. Optimally, automated software for JSW/mJSW-detection and measurement would be used in such a study^17^ but was not feasible in this study. Measurement of the cartilage thickness on each bone surface would be a better method of assessing the correct total cartilage thickness in each compartment, but this was not possible in knees with very deteriorated cartilage. Therefore, MRi measurements from cortex to cortex of both JSW/mJSW were considered the best measurement method in this study to compare with radiographic JSW/mJSW. The knee joint was not in a weight-bearing position when performing MRi, theoretically allowing the possibility of fluid to enter the compartment between the joint surfaces, but this was not an actual issue during measurements. In some cases, this could increase the measured JSW/mJSW, but earlier studies have shown this to have a marginal impact.^9^ The knee was positioned in different degrees of flexion in each method, but this reflects general clinical practice.

## 5 Conclusions

Non-significant weak correlation was found between specialized radiography and MRi when measuring cartilage height in the medial knee compartment. A strong to very strong correlation was found between specialized radiography and MRi in the lateral compartment. We conclude that MRi cannot and should not replace these specialized radiographic methods but should be reserved for more special cases where abnormal radiography or suspicion of atypical clinical findings present themselves.

## Ethical policies

Participants gave informed consent before inclusion, and the study has obtained approval from the Danish Ethics Committee, approval-id H-18010291.

There were no financial conflicts of interest. The authors' institutions funded the study without external funding.
